# Supplementary material for: Splice-Junction-Based Mapping of Alternative Isoforms in the Human Proteome
Source: Cell Rep. Author manuscript; Available in PMC 2020 Jan 15. (PMC6961840; doi:10.1016/j.celrep.2019.11.026)

A

sp|P09382|LEG1_HUMAN|ENSG00000100097|SE1|5858|chr22|37677065|37678392|+2|r860|T4
GGSLNLKPGECLR q value: 0.0010669 Tr_novel:TRUE RefSeq_Novel:TRUE
Search result spec prec mz: 700.871 Actual spec prec mz: 700.87103
Fragments matched per AA: 2.08 Proportion of top 20 peaks matched: 0.5

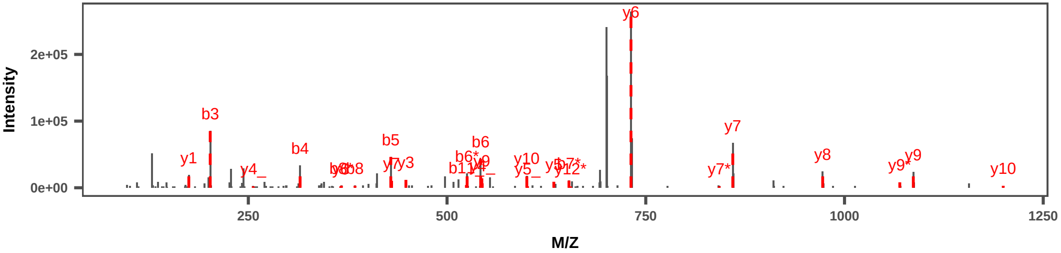

B

Scatterplot of predicted elution time
Fitting R2: 0.791
Novel peptide residual Z score: −0.765
Number of peptides: 1946

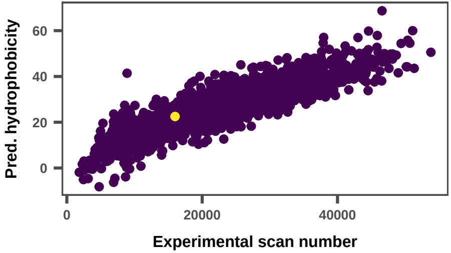

C

Distributions of residuals from best−fit line
of predicted RT vs Expt. scan number
Line: Z score of novel peptide
Z: −0.765

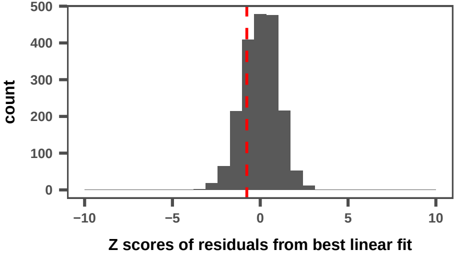

Supplement: 2 [file NIHMS1546469-supplement-2.zip › DF1/PXD010154/Esophagus/Esophagus_14_LGALS1_GGSLNLKPGECLR.pdf]
